# Total Esophageal Avulsion at the Esophagogastric Junction after Blunt Trauma

**DOI:** 10.1155/2013/265073

**Published:** 2013-04-08

**Authors:** Ibrahim Uygun, Selcuk Otcu, Bahattin Aydogdu, Mehmet Hanifi Okur, Mehmet Serif Arslan

**Affiliations:** Department of Pediatric Surgery, Medical Faculty of Dicle University, 21280 Diyarbakir, Turkey

## Abstract

Total avulsion and transection of the esophagus at the esophagogastric junction are very rare after blunt trauma, and their management is challenging. Here, we present the case of a boy with this injury. To date, only two cases have been reported in children. One was treated successfully and the other died. The initial emergency operation should aim to save the life and native esophagus. Therefore, a primary or early thoracal end esophagostomy with gastrostomy should be performed, while primary repair should not be.

## 1. Introduction

The frequency of traumatic esophageal injures varies by country. However, they occur in <1% of all traumatic injuries but are associated with a significant risk of morbidity and mortality (6%–70%) [1, 2]. The outcome of esophageal injuries is determined by several factors that increase the risk of complications and death. A delay in diagnostic studies to determine the presence of these injuries and the difficulty identifying these injuries, particularly when other life-threatening injuries are present, can be problematic [1, 2]. Late surgical intervention for esophageal injuries is the most important determinant of the high rates of mortality and morbidity [1, 2].

Total avulsion and transection of the esophagus at the esophagogastric junction are very rare after blunt trauma, and their management its challenging. To-date, only two cases have been reported in children [3, 4]. Here, we present the case of a boy with this injury.

## 2. Case Report

**Figure 1:**
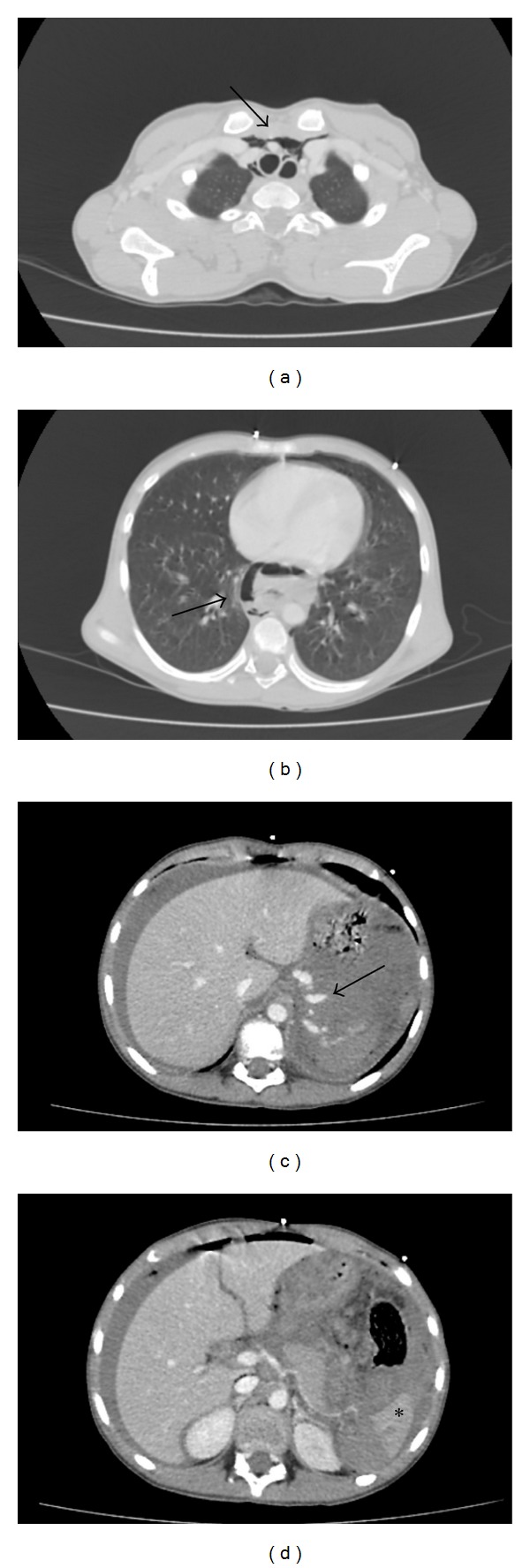
Thoracoabdominal emergency computerized tomography. (a) and (b) Mediastinal massive free air (arrows). (c) and (d) Laceration of the spleen (asterisk), massive intra-abdominal free fluid with free air, and active intraperitoneal bleeding (arrow).

**Figure 2:**
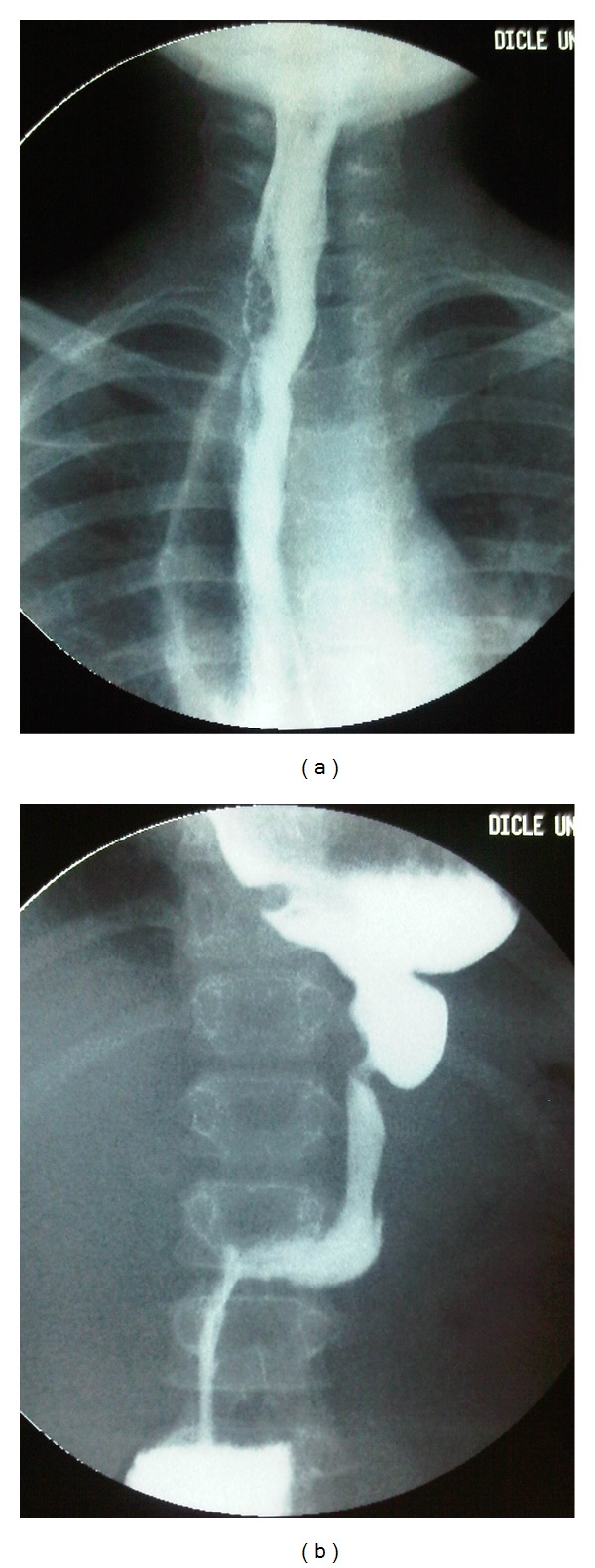
Barium swallow study after a gastric pull-up procedure. (a) Cervical esophagogastrostomy and stomach in the mediastinum. (b) Pylorus and duodenum in the abdomen.

A 12-year-old boy was admitted to the emergency department due to a bicycle accident. A car had hit him 30 min previously. He was in hypovolemic shock (Glasgow coma score 10), and he had an acute abdomen with abdominal distention. An emergency thoracoabdominal computerized tomography with intravenous contrast agent showed laceration of the spleen (grade V), intra-abdominal massive free fluid with free air, active intraperitoneal bleeding, mediastinal massive free air, and multiple pubic fractures (Figure 1). He underwent an emergency exploratory laparotomy. During the operation, we discovered massive intra-abdominal blood with gastric contents (food particles, chips, etc.) and a splenic laceration with active bleeding. Thus, a splenectomy was performed. The stomach was ruptured on the anterior corporeal wall at approximately 8 cm, and the stomach was totally free. A total esophageal avulsion at the esophagogastric junction and active bleeding from the esophageal hiatus were identified. The avulsed distal esophageal end was ragged (tassel-like) and had retracted into the mediastinum. Gastric contents were present in the mediastinum. No other intra-abdominal injuries were noted. After the bleeding was controlled and washed out, the gastric rupture was repaired. An esophagogastrostomy at the site of the avulsion was carried out over a nasogastric tube after mobilizing the esophageal distal end. Two drains were inserted: one into the lower abdomen and the other into the mediastinum at the esophagogastric junction. After the operation, he was mechanically ventilated for 24 hours. He was fed on postoperative day four via a nasojejunal tube, because there was no discharge from the drains. Nevertheless, he was operated on for a second time for a thoracotomy due to uncontrolled mediastinitis signs (severe fever, tachypnea, and tachycardia) on postoperative day 13, and mediastinitis due to an esophagogastric anastomotic leakage was identified. A mediastinal tube thoracostomy drainage and esophageal repair were performed. However, the esophageal leakage and severe mediastinitis could not be controlled by several sequential interventions (esophageal end closure and gastrostomy, cervical loop esophagostomy, and esophageal stapling with a stapler). Esophageal leakage of saliva and mediastinal discharge continued, from which bacteria (*Acinetobacter baumannii*, *Cedecea lapagei*, and *Tatumella ptyseos*) were isolated. These were suppressed successfully with broad-spectrum antibiotics such as cefoperazone-sulbactam, linezolid, and tigecycline. The patient was fed fully via the gastrostomy. Finally, right lateral cervical end esophagostomy was performed. Subsequently, he improved quickly and was discharged uneventful with esophagostomy and gastrostomy. Six months after the accident, a gastric pullup was performed to replace the esophagus. A postoperative anastomotic leakage was treated medically with antibiotics, tube thoracostomy, and nasojejunal feeding, and he was discharged uneventful. After esophageal replacement surgery, he is well and can swallow solid food without dysphagia at the two-year followup (Figure 2).

## 3. Discussion

Esophageal injuries are rare because the elasticity, mobility, and secluded position of the esophagus protect it from blunt trauma; however, if injured, management of an esophageal injury is a challenge for the surgeon [1, 2]. Esophageal leakage is the main problem, as it may cause mediastinitis, pneumonia, sepsis, or death [1, 2]. Treatment of esophageal leakage after failure of an anastomosis and iatrogenic perforation is mostly medical, but after traumatic injury, it is mostly surgical [1, 2]. Saliva and gastric acid contents may leak after opening the esophagus. If the diagnosis is delayed, fluid and solid food may leak. Early treatment is the most important factor for morbidity and mortality due to esophageal damage [1, 2]. However, management of a total avulsion and transection of the esophagus at the esophagogastric junction is very challenging for the surgeon even if it is diagnosed early. Data are insufficient, as only two cases of transection of the esophagus at the esophagogastric junction by blunt trauma have been reported in children [3, 4].

In the first case reported by Barrie et al. in 1961, a 13-year-old girl sustained a fall on the left chest and abdomen [3]. Primary reconstruction was performed by esophagogastrostomy through separate abdominal and thoracic incisions. Hypotension, hyperthermia, and renal shutdown developed postoperatively, and she died four days after the injury. The esophageal reconstruction was intact at autopsy. The authors questioned the advisability of a prolonged primary reconstruction versus a shorter exclusion operation that carried the disadvantage of a cervical esophagostomy [3].

In the second case reported by Miller in 1968, a 13-year-old boy was in a truck accident and was operated on via an extended laparothoracotomy [4]. After a left mediastinal and thoracal washout, the authors closed the proximal end of the esophagus rather than risk the chance of an anastomotic leakage from a primary esophagogastric reconstruction; thus, a cervical double-barrel esophagostomy to defunctionalize the thoracic esophageal segment was performed. However, purulent discharge from the posterior mediastinal tube drain continued for six weeks, and the patient developed recurrent suppurative mediastinitis. Then, an esophagography showed free communication of the distal end of the esophagus with the mediastinum, which was believed to be the source of the recurrent mediastinal contamination. He recovered promptly after an esophagectomy. Esophageal replacement surgery was conducted using the terminal ileum and right colon. A minimal leak at the proximal anastomosis closed after one week. Finally, he was well. This was the first reported case of recovery from an esophageal avulsion [4].

In our case, we tried to save the life and the esophagus. But, we could not save all of the native esophagus. We did insert effective drains into the abdomen and mediastinum and a feeding tube such as nasojejunal tube or gastrostomy/gastrojejunal tube to supply energy. An esophagogastrostomy or closure of the proximal end of the esophagus to save the esophagus has been tried, but all failed. The infection caused by the contamination and presence of food particles in the mediastinum, even if they were washed out, was the most important factor. Infection impairs wound healing and also tissue damage, necrosis, and loss of the end of avulsed organs. Tissue-loss-induced tension from the anastomosis causes leakage, which leads to infection. Additionally, esophageal mucosal secretions are important. Because the esophagus always secretes mucosal secretion, which collects in the esophageal pouch when the esophagus is closed blindly, even if the saliva is stopped using cervical loop or a double-barrel esophagostomy [4, 5]. The collection of secretions in the esophageal pouch causes infection and leakage [4]. Furthermore, esophageal motility together with the secretions opens the closure in the esophagus as in our case and in the second case reported by Miller [4].

Mediastinitis caused by esophageal leakage is the most important reason for death after esophageal injury and must be prevented or controlled [1, 2]. Therefore, a primary or early thoracal end esophagostomy should be performed to save the life and as much as possible the native esophagus, but primary repair should not be attempted in cases of the total esophageal avulsion at the esophagogastric junction [4].

Antibiotic-resistant bacterial nosocomial infections are a major problem, particularly for patients with prolonged hospitalization in the intensive care unit. *A. baumannii *is the most significant nosocomial infection agent in our country and in our hospital [6]. It was isolated from the mediastinal discharge and was susceptible to cefoperazone/sulbactam, piperacillin/tazobactam, and tigecycline only but was uniformly resistant to other antimicrobial agents. Interestingly, *C. lapagei* and *T. ptyseos*, which are very uncommon agents, were also isolated and were susceptible to cefoperazone/sulbactam [7, 8].

Managing a total avulsion and transection of the esophagus at the esophagogastric junction after blunt trauma is challenging for the surgeon. The initial emergency operation should aim to save the life and native esophagus. Therefore, a primary or early thoracal end esophagostomy with gastrostomy should be performed, while primary repair should not be.
